# Estimated Effects of Projected Climate Change on the Basic Reproductive Number of the Lyme Disease Vector *Ixodes scapularis*

**DOI:** 10.1289/ehp.1307799

**Published:** 2014-03-14

**Authors:** Nicholas H. Ogden, Milka Radojevic´, Xiaotian Wu, Venkata R. Duvvuri, Patrick A. Leighton, Jianhong Wu

**Affiliations:** 1 Zoonoses Division, Centre for Food-borne, Environmental and Zoonotic Infectious Diseases, Public Health Agency of Canada, Saint-Hyacinthe, Quebec, Canada; 2 Centre for Disease Modelling, York Institute of Health Research, Toronto, Ontario, Canada; 3 Department of Mathematics and Statistics, York University, Toronto, Ontario, Canada; 4 Faculty of Veterinary Medicine, Université de Montréal, Saint-Hyacinthe, Quebec, Canada; * These authors are equal first authors.

## Abstract

Background: The extent to which climate change may affect human health by increasing risk from vector-borne diseases has been under considerable debate.

Objectives: We quantified potential effects of future climate change on the basic reproduction number (*R*_0_) of the tick vector of Lyme disease, *Ixodes scapularis*, and explored their importance for Lyme disease risk, and for vector-borne diseases in general.

Methods: We applied observed temperature data for North America and projected temperatures using regional climate models to drive an *I. scapularis* population model to hindcast recent, and project future, effects of climate warming on *R*_0_. Modeled *R*_0_ increases were compared with *R*_0_ ranges for pathogens and parasites associated with variations in key ecological and epidemiological factors (obtained by literature review) to assess their epidemiological importance.

Results: *R*_0_ for *I. scapularis* in North America increased during the years 1971–2010 in spatio-temporal patterns consistent with observations. Increased temperatures due to projected climate change increased *R*_0_ by factors (2–5 times in Canada and 1.5–2 times in the United States), comparable to observed ranges of *R*_0_ for pathogens and parasites due to variations in strains, geographic locations, epidemics, host and vector densities, and control efforts.

Conclusions: Climate warming may have co-driven the emergence of Lyme disease in northeastern North America, and in the future may drive substantial disease spread into new geographic regions and increase tick-borne disease risk where climate is currently suitable. Our findings highlight the potential for climate change to have profound effects on vectors and vector-borne diseases, and the need to refocus efforts to understand these effects.

Citation: Ogden NH, Radojević M, Wu X, Duvvuri VR, Leighton PA, Wu J. 2014. Estimated effects of projected climate change on the basic reproductive number of the Lyme disease vector *Ixodes scapularis*. Environ Health Perspect 122:631–638; http://dx.doi.org/10.1289/ehp.1307799

## Introduction

Considerable attention has been devoted to the possibility that climate change will exacerbate the burden of mosquito-borne diseases such as malaria and dengue, with important impacts on public health (Githeko et al. 2000). Early assessments of the effects of climate change on malaria and dengue used simplistic models to assess possible effects of climate change on their basic reproductive numbers (*R*_0_, the universally recognized metric of the capacity of a parasite or pathogen to reproduce given particular environmental conditions) (Martens et al. 1995; Patz et al. 1998). However, these assessments were criticized for giving weight to future increases in *R*_0_ whether or not such increases resulted in *R*_0_ rising above the critical threshold of > 1 for disease persistence (Rogers and Randolph 2000) and for being oversimplistic by only accounting for climate effects rather than the full range of nonclimatic factors that impact the occurrence of these diseases (Reiter 2001; Rogers and Randolph 2000). Any impact of climate on *R*_0_ of malaria and dengue is limited by the effects of variations in human host density, mosquito control, infection prevention and treatment in humans, and human management of the environment (e.g., agriculture, forest management, logging) that affect the ecology and epidemiology of the vectors, pathogens, and diseases (Githeko et al. 2012). Consequently, the strength of evidence for recent climate warming effects on malaria risk has been questioned and much debated (Reiter et al. 2004; Tanser et al. 2003).

Many vector-borne diseases of public health significance (e.g., Lyme disease, West Nile virus) are, however, maintained in transmission cycles that involve wild animal hosts. These cycles are independent of human cases, and the spatio-temporal risk of human disease is less dependent on the direct effects of human activities than is the risk from malaria and dengue. Nevertheless, despite some assessments (Gubler et al. 2001), the effects of climate change on vector-borne zoonoses have also been downplayed mostly on the basis of limited evidence for recent effects of climate change (Kilpatrick and Randolph 2012). Lyme disease emerged (or likely reemerged) in the northeastern United States in the late 1970s due to the expansion of tick populations, which was generally thought to have been associated with changes in land use over some decades that resulted in reforestation and expansion of the population of the deer that are key hosts for the ticks (Wood and Lafferty 2013). Lyme disease is now emerging in Canada and some northern U.S. states due to the northward expansion of the geographic range of the tick vector *Ixodes scapularis* (*I. scapularis*) (Hamer et al. 2010; Ogden et al. 2009), which is dispersed from source populations by migratory birds and terrestrial hosts (Leighton et al. 2012).

A mechanistic simulation model of the *I. scapularis* life cycle has identified temperature effects on *I. scapularis* population survival in order to assist in assessment of current and future on-the-ground Lyme disease risk in Canada (Ogden et al. 2005, 2006b). Prospective field studies and retrospective analyses of surveillance data on tick and pathogen emergence in southeastern Canada validated the model findings and identified temperature as a statistically significant determinant and possible driver of emergence of the tick in Canada (Bouchard et al. 2013a, 2013b; Leighton et al. 2012; Ogden et al. 2008, 2010). The *I. scapularis* model was modified to permit the direct calculation of *R*_0_ for *I. scapularis* via the next generation operator approach (Wu et al. 2013), which, given the universal use of *R*_0_ and its estimation for a wide range of parasites and pathogens under many different conditions, allowed comparison of *R*_0_ variations in the present study with observed variations for other parasites and pathogens.

Here, we have estimated projected effects of climate change on *R*_0_ of an arthropod vector using a model that has been extensively ground-truthed, and we have assessed the ecological and epidemiological significance of the projected changes in *R*_0_ by comparing them with ranges of *R*_0_ values observed for other parasites and pathogens.

## Methods

We estimated *R*_0_ under current and future projected climatic conditions at 30 sites in Canada that formed two roughly south–north transects in Ontario and Quebec, two Canadian provinces where *I. scapularis* ticks are becoming established. These transects were chosen to capture the climate variability that exists in the region. For simplicity in data presentation, the sites were grouped into clusters [Southern Ontario, Huron Ontario, Upper Southern Ontario, South-Western Quebec, and the Boreal region (see Supplemental Material, Table S1 and Figure S1)] according to geographic proximity and similarity in temperature conditions (see Supplemental Material, “Variation in temperature and *R*_0_ amongst sites,” pp. 2–6, Figures S2–S4), and mean values for clusters are presented. We also estimated *R*_0_ for two sites in the United States where Lyme disease is endemic in the Northeast and upper Midwest, respectively: Old Lyme (Connecticut), where the human Lyme disease cases were first recognized (Wood and Lafferty 2013), and Fort McCoy (Wisconsin) (Anderson et al. 1987).

*Modeling* R*_0_*_._ The *I. scapularis* model is a deterministic model consisting of 12 ordinary differential equations as described by Wu et al. (2013), based on the mechanistic simulation model described by Ogden et al. (2005). This model captures the effects of temperature on host-seeking activity and the rates of development from one life stage to the next (effects common to, but variable among, all arthropod vectors), parameterized from field and laboratory studies on *I. scapularis*. Mortality rates of nonfeeding *I. scapularis* in Canada and the northeastern United States are similar in summer and winter, presumably due to the insulating effects of the litter layer in woodland habitats (Brunner et al. 2012; Lindsay et al. 1995). Our analyses operated on the hypothesis that the effects of ambient temperature on *I. scapularis* population survival are indirect via effects on temperature-dependent rates of development of ticks from one life stage to the next. The lower the temperature, the longer is the tick life cycle and, due to constant daily per capita mortality, the fewer larval ticks survive to become mated adult female ticks. At a threshold temperature, mortality outstrips reproduction and the tick populations die out (or fail to become established)—that is, at this temperature, threshold *R*_0_ falls below unity (Ogden et al. 2005). At the latitudes under study here, effects of climate change on *I. scapularis* are expected to be the effect of climate warming on shortening the life cycle, resulting in increasing *R*_0_ (Ogden et al. 2006b). Quadratic effects of temperature on arthropod vector life history traits are common (Mordecai et al. 2013), and quadratic effects of temperature on tick activity are included in the model. High temperatures may impact tick survival, causing a northward contraction of the southern range of *I. scapularis*, resulting in a northward shift, rather than overall expansion, of the geographic range of climatic suitability for *I. scapularis* (Brownstein et al. 2005). Here we confined our study to Canada and the main regions of Lyme disease risk in the United States north of 40°N (Diuk-Wasser et al. 2012). Impacts of rainfall on off-host tick survival and on host-seeking activity are considered accounted for in the model in assuming *a*) tick populations only become established in woodlands where the microclimate is suitable for tick survival, and *b*) most temperate woodlands types occur where rainfall is sufficient for *I. scapularis* survival, which is supported by studies in Canada (Lindsay et al. 1995). Future projections for increased precipitation across much of Canada with climate warming are already being seen to occur (Environment Canada 2013), so rainfall changes are not expected to limit the northward spread of *I. scapularis*. For the present study, we modified the simulation model of Ogden et al. (2005) in order to calculate *R*_0_ by the next generation operator as described by Wu et al. (2013). Apart from the temperature values used to calculate tick development and host-finding rates, the values for host numbers (20 deer and 200 rodents) and all other parameter values were those used as starting values by Wu et al. (2013). *R*_0_ was estimated using mean monthly temperature data for each year and location as described in the following sections. Variations in host abundance affect the final size of the tick population but not the temperature threshold (Ogden et al. 2005). The temperature threshold would be affected by variations in the mortality rates of ticks in the environment, and slight variations in this have been observed in the field (Ogden et al. 2006a). For the sensitivity analysis of *R*_0_ to variations in model parameter values, see Supplemental Material, “Model sensitivity analysis,” pp. 7–11, Table S2 and Figures S5 and S6.

*Modeling* R*_0_ under current climate*. For observed temperatures, we used Australian National University Splines (ANUSPLIN) (Hutchinson et al. 2009) of 10-km gridded daily time-series data, which were obtained by thin-plate smoothing spline interpolation of daily climate station observations while accounting for latitude, longitude, and elevation. ANUSPLIN data cover the 40 years (1971–2010) that encompass the period of Lyme disease emergence in North America, have coverage across northern North America, and account for missing data by temporal and spatial interpolation. A mean daily near-surface temperature was assigned to the 32 study sites, which were weather stations located over a wide range of the orographic and forest ecosystems of Ontario and Quebec or interpolations of ANUSPLIN data for locations in the United States. Monthly mean near-surface air temperatures were used to parameterize the *I. scapularis* population model for estimating annual values of *R*_0_ for each site, for each year from 1971 to 2010 (see Supplemental Material, “Variation in temperature and *R*_0_ amongst sites,” pp. 2–6). Values for annual cumulative degree days > 0°C (DD > 0°C), contemporaneous for each estimated *R*_0_ value, were computed as the accumulation of daily temperature > 0°C for each year for each site. The tick model calculated *R*_0_ for each year using temperature data for that year, but in reality *R*_0_ will depend on the temperature conditions over the 2- to 3-year life cycle of the tick. Therefore, we used the moving average of *R*_0_ over 3 years (that year, the previous year, and the subsequent year) to describe *R*_0_ for each year for each site; so for each site, we obtained a time series for *R*_0_ and DD > 0°C for 38 years (1972–2009) and DD > 0°C for 40 years (1971–2010).

*Modeling* R*_0_ using projected climate data*. An ensemble of modeled temperature data available from three regional climate model (RCM) and two global climate model (GCM) runs were used to estimate future changes in *R*_0_ (see Supplemental Material, “Validation of climate model output,” pp. 11–14; Table S3). We extracted time series of daily temperature for the 30 Canadian sites from each model. Simulated temperature at a given site was defined as the mean of the closest grid values to that site, increasing confidence in the physical representativeness (Gachon and Dibike 2007).

We chose bias-corrected output from Canadian RCM CRCM4.2.3, version 4 (Laprise et al. 1997; Music and Caya 2007) to provide temperature data for the *I. scapularis* model because it relatively accurately and conservatively hindcasted observed ANUSPLIN data in comparison with other climate models and because it provided projected temperatures at a local scale. For full details justifying the climate model selection, see Supplemental Material, “Validation of climate model output,”pp. 11–14, Figures S7 and S8. Like other RCMs, CRCM4.2.3 dynamically downscales output from a coarser resolution GCM and produces data at a horizontal resolution of approximately 50 km. CRCM4.2.3 is driven by initial and boundary conditions of the Canadian GCM CGCM3.1 T47 (McFarlane et al. 2005; Scinocca et al. 2008). Hindcasting up to 2000 used greenhouse gas emissions for CGCM3.1 T47 as in the Coupled Model Intercomparison Project (CMIP) 20th century experiment (Meehl et al. 2000). For future projections starting in 2001, we chose the A2 scenario (mid-high Green-House-Gas emission scenario) of the Intergovernmental Panel on Climate Change *Special Report on Emission Scenarios* (Nakicenovic and Swart 2000) because of the availability of regional climate model output using this scenario and because current actual trajectory of emissions corresponded best to this emissions scenario.

**Figure 2:**
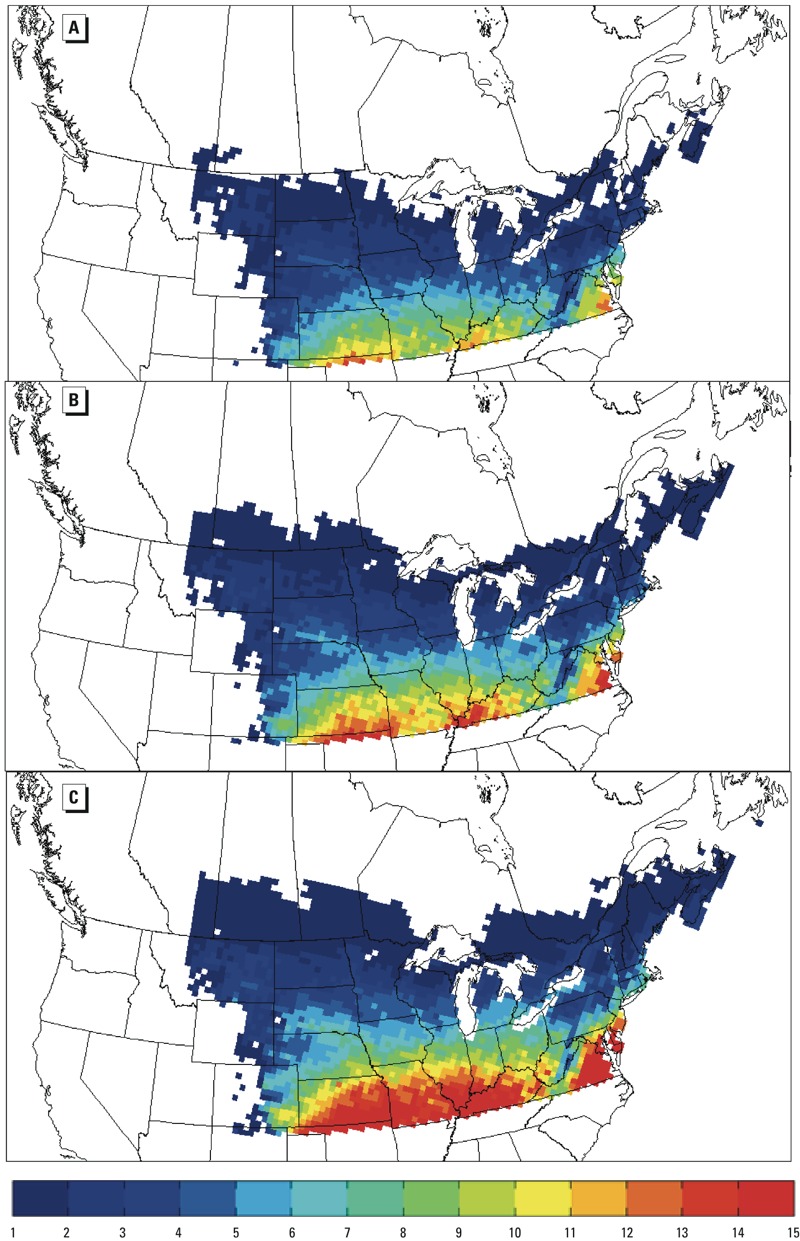
Maps of values of *R*_0_ estimated from ANUSPLIN observations (1971–2000; *A*) and projected climate obtained from the CRCM4.2.3 driven by CGCM3.1 T47, following the SRES A2 GHG emission scenario for 2011–2040 (*B*) and 2041–2070 (*C*). The color scale indicates *R*_0_ values. Within the zones where *R*_0_ of *I. scapularis* is > 1, geographic occurrence of Lyme disease risk is also limited by other environmental variables (Diuk-Wasser et al. 2012).

*Mapping* R*_0_ under current and future climate*. We mapped 30-year mean values of *R*_0_ for *I. scapularis* using observed and projected values. Maps of DD > 0°C for North America north of 40°N and east of the Rocky Mountains were generated using observed data for 1971–2000, and using DD > 0°C projected by bias-corrected CRCM4.2.3 output for the period 2001–2070. We then computed *R*_0_ for each year from the gridded DD > 0°C data using the formula *R*_0_ = 1.072 × 10^–6^ DD > 0°C–4.658 × 10^–3^ DD > 0°C^2^ + 5.556, obtained using observed temperature data as described in Supplemental Material, “Mapping *R*_0_,”pp. 15–16, Figure S9 (the threshold for *R*_0_ ≥ 1 was DD > 0°C ≥ 2859.6°C). We then mapped mean 30-year values for *R*_0_ for the periods 1971–2000, 2011–2040, and 2041–2070 (Figure 2). Regions west of the Rocky Mountains were masked because it was assumed that *I. scapularis* will not cross the Rocky Mountains, west of which Lyme disease risk will continue to depend on transmission of *Borrelia burgdorferi* by the tick *I. pacificus* (Ogden et al. 2009).

*Literature search on* R*_0_ ranges for parasite and pathogen systems*. There are, to our knowledge, no equivalent estimates of how environmental changes may affect *R*_0_ of vectors. However, to better comprehend the ecological or epidemiological importance of projected changes of *R*_0_ of *I. scapularis*, we performed a literature review to obtain published estimates of how *R*_0_ for parasites and pathogens varies due to changes in factors already recognized as having ecological or epidemiological importance. These include variations in geographic location, host density, strain or genotype, disease control effort, and variations among different epidemics. Articles were searched in the National Centre for Biotechnology Information PubMed search site (http://www.ncbi.nlm.nih.gov/pubmed/) using search terms *a*) “basic reproduction number,” without specifying pathogens or parasites, and then *b*) “basic reproductive number,” repeated with one of the following terms: tick, mosquito, chagas, malaria, dengue, nematode, “seasonal influenza,” “pandemic influenza”, pH1N1, “avian influenza,” measles, HIV (human immunodeficiency virus), and fluke. Abstracts were reviewed, and relevant articles were reviewed in full. Relevant articles were those in which *R*_0_ for parasites and pathogens was calculated to explicitly estimate its value under field, rather than theoretical, conditions. This meant articles that employed simulation models using field data, fitting of epidemiological data (e.g., age–seroprevalence or age–infection prevalence), or other methods such as estimates from phylogenetic analysis. We did not review and use *R*_0_ ranges obtained in model-based sensitivity analyses, variations in *R*_0_ associated with seasonal variations in mosquito abundance in one location (which may vary from zero to very high values), estimates where control methods effectively eradicated disease resulting in almost infinite values for changes in *R*_0_, or model-predicted variations across whole potential geographic ranges that range from a theoretical high to zero values (e.g., Estrada-Peña et al. 2013). We also did not use some very high modeled ranges of *R*_0_ for malaria [e.g., 1–11,000 (Smith et al. 2007)] when the modeling of empirical age–infection prevalence data produced strongly contrasting single-digit estimates of *R*_0_ (Hagmann et al. 2003; Hay et al. 2005). The goal of the literature search was to provide an illustration of how important projected changes in *R*_0_ of *I. scapularis* could be, compared with *R*_0_ of other parasites and pathogens; it was not intended to be an exhaustive cataloging of all literature in this field. Further, we recognized that *R*_0_ estimates are not precise and vary according to the estimation method used (Heffernan et al. 2005; Li et al. 2011).

## Results

**Figure 1:**
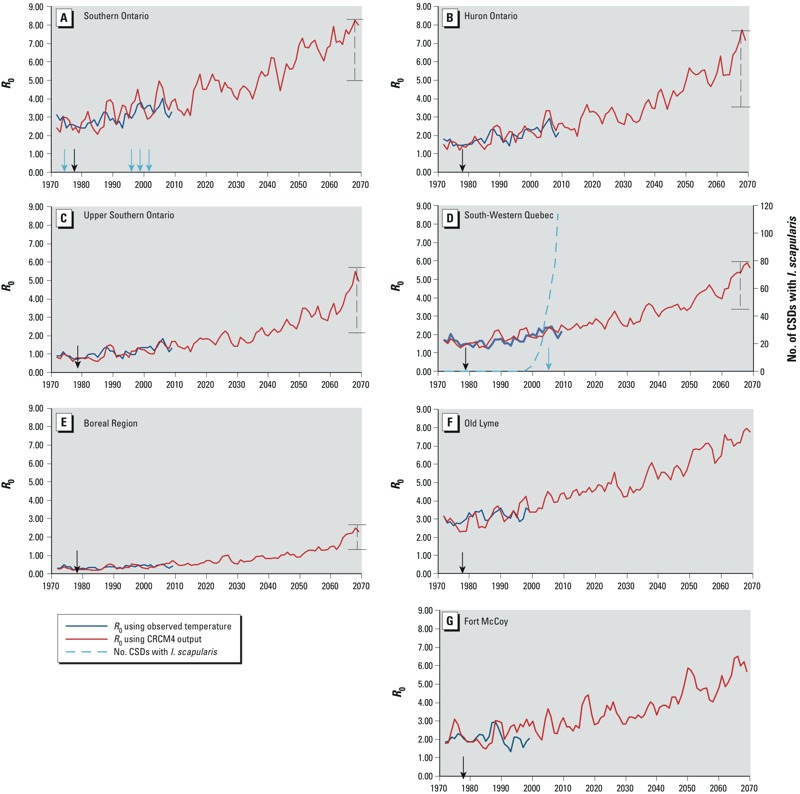
Mean values for *R*_0_ of the tick *I. scapularis* obtained in tick model simulations using observed temperature data (ANUSPLIN: 1971-2010), and projected temperature data obtained from the RCM CRCM4.2.3 [according to Special Report on Emissions Scenarios (SRES) A2 emissions scenario] for (*A*) Southern Ontario, (*B*) Huron Ontario, (*C*) Upper Southern Ontario, (*D*) South-Western Quebec, (*E*) the Boreal region of central Ontario and Quebec, (*F*) Old Lyme (Connecticut), and (*G*) Fort McCoy (Wisconsin). The black arrows in each panel reference the first identification of Lyme disease in the United States (Wood and Lafferty 2013). The green arrows indicate the year of first field detection of *I. scapularis* populations within the Canadian clusters. (*A*) In Southern Ontario, these dates are 1976 for Long Point (Watson and Anderson 1976), 1996 for Point Pelee (Lindsay et al. 1999a), 1999 for Rondeau Park (Morshed et al. 2003), and 2001 for Turkey Point (Scott et al. 2004). (*D*) The date is 2007 for a number of sites in South-Western Quebec (Ogden et al. 2008); the estimated numbers of Census Subdivisions (CSDs) with established *I. scapularis* populations in South-Western Quebec, based on passive surveillance data (Leighton et al. 2012), is shown as the green dashed line. The range of *R*_0_ values produced in simulations for 2020–2069 of CRCM4.2.3 and five other GCMs and RCMs is indicated by the error bar to the right of each panel except for the U.S. sites (*F,G*), for which only output from CRCM4.2.3 was available. Full details of all simulations are presented in Supplemental Material, Figure S6.

Using observed (ANUSPLIN) temperature data, *R*_0_ for *I. scapularis* in the late 1970s—when Lyme disease emerged in the northeastern United States (Wood and Lafferty 2013)—was estimated at approximately 3 and 1.9 in Old Lyme and Fort McCoy, respectively; at between 2 and 3 in Southern Ontario; approximately 1.5 in Huron Ontario and South-Western Quebec, but mostly < 1 in Upper Southern Ontario and the Boreal region (Figure 1). In Old Lyme, *R*_0_ increased almost linearly to approximately 3.5 by 1999 during the first period of expansion of *I. scapularis* in the northeastern United States. In Fort McCoy, *R*_0_ increased slightly, but this increase was small compared with interannual variations. In Southern Ontario, *R*_0_ increased to 4 by the early 2000s; during which time *I. scapularis* populations emerged at a number of locations in this region (Point Pelee National Park, Turkey Point, Rondeau Provincial Park; Figure 1A). In Huron Ontario and South-Western Quebec, *R*_0_ increased from 1.5 to 2.5 by the early 2000s; and subsequent to this (mostly from 2000 onward), *I. scapularis* populations began to emerge in South-Western Quebec (Figure 1D). In Upper Southern Ontario, *R*_0_ increased to > 1 in the late 1990s, but in the Boreal region *R*_0_ remained below unity for the whole 1971–2010 period (Figure 1).

*R*_0_ values for *I. scapularis* obtained in model simulations using projected climate data were similar for an ensemble of climate models, and we used bias-corrected output from the regional climate model CRCM4.2.3 as a representative of the ensemble because of its spatial resolution and predictive accuracy. *R*_0_ for *I. scapularis* in Canada was projected to increase 1.5 to 2.3 times from the period 1971–2000 to 2001–2050, and 2.2 to 4.6 times from the period 1971–2000 to 2051–2069 depending on location (Figure 1, Table 1); and in the United States, *R*_0_ was predicted to approximately double to 7.1 and 5.2 in Old Lyme (Figure 1) and Fort McCoy, respectively, by 2051–2069 (Table 1). Increases in *R*_0_ to values > 1 predicted in regions where *R*_0_ was < 1 during the period 1970–2000 would be expected to facilitate range expansion of *I. scapularis* northward and possibly westward (Figure 2).

**Table 1:** *R*_0_ values quantified for infectious diseases, arthropod vectors, and vector-borne diseases.

Pathogen or parasite	*R*_0_ range estimate	Factors associated with variation	References
Directly transmitted infectious diseases
Cholera in Zimbabwe	1–2.72 (× 2.7)	Environment, socio­economic conditions, and cultural practices	Mukandavire et al. 2011
1918–1919 A/H1N1 Pandemic influenza	1.5–7.5 (× 5)	Human population density	Chowell et al 2007; Massad et al. 2007; Mills et al. 2004; Vynnycky et al. 2007
1957–1958 A/H2N2 Pandemic influenza	1.4–1.7 (× 1.2)	Country	Longini et al. 2004; Nishiura 2010b
2009 A/H1N1	1.3–1.7 (× 1.4)	Country, community, human population density	Fraser et al. 2009; Pourbohloul et al. 2009; Tuite et al. 2010; White et al. 2009; Yang et al. 2009
Low-pathogenic influenza A viruses in turkey flocks	0.6–5.5 (× 9.2)	Virus strain and farm	Comin et al. 2011
H5N1 influenza A in poultry	1–3 (× 3)	Different global epidemics	Zhang et al. 2012
H7N7 influenza A in poultry	1.2–6.5 (× 5.4)	With and without control	Stegeman et al. 2004
Seasonal influenza	1.6–3 (× 1.9)	Locations, years, and viral strains	Gran et al. 2010; Truscott et al. 2012
HIV (human immunodeficiency virus)	1.1–3.7 (× 3.4)	Country and subepidemic	Nishiura 2010a; Stadler et al. 2012; Xiao et al. 2013
SARS (severe acute respiratory syndrome)	1.2–8 (× 6.7)	Modeling methods and human population demography	Bauch et al. 2005
Measles	1.2–9.5 (× 7.9)	Vaccination, different schools	Mossong and Muller 2000; Plans Rubio 2012
Polio	2–14 (× 7)	Levels of hygiene	Fine and Carneiro 1999
Canine rabies	1.05–2.44 (× 2.3)	Location across the world	Fitzpatrick et al. 2012; Kitala et al. 2002
African swine fever	2–3 (between farms: × 1.5), and 8–11 (within farms: × 1.4)	Location in Russian federation	Gulenkin et al. 2011
Foot and Mouth disease in cattle	1.6–4.5 (× 2.8)	With and without control	Ferguson et al. 2001
Arthropod vectors: *Ixodes scapularis* in Canada
Boreal region, 1971–2000 vs. 2001–2050	0.3–0.7 (× 2.3)	Climate change	This study
Boreal region, 1971–2000 vs. 2051–2069	0.3–1.4 (× 4.6)	Climate change	This study
Huron Ontario, 1971–2000 vs. 2001–2050	1.8–3.0 (× 1.6)	Climate change	This study
Huron Ontario, 1971–2000 vs. 2051–2069	1.8–5.3 (× 2.9)	Climate change	This study
Southern Ontario, 1971–2000 vs. 2001–2050	3.0–4.5 (× 1.5)	Climate change	This study
Southern Ontario, 1971–2000 vs. 2051–2069	3.0–6.7 (× 2.2)	Climate change	This study
Upper Southern Ontario, 1971–2000 vs. 2001–2050	0.9–1.7 (× 1.9)	Climate change	This study
Upper Southern Ontario, 1971–2000 vs. 2051–2069	0.9–3.3 (× 3.6)	Climate change	This study
South-Western Quebec, 1971–2000 vs. 2001–2050	1.7–2.8 (× 1.6)	Climate change	This study
South-Western Quebec, 1971–2000 vs. 2051–2069	1.7–4.3 (× 2.5)	Climate change	This study
Old Lyme, CT, USA, 1971–2000 vs. 2001–2050	3.1–4.8 (× 1.5)	Climate change	This study
Old Lyme, CT, USA, 1971–2000 vs. 2051–2069	3.1–7.1 (× 2.3)	Climate change	This study
Fort McCoy, WI, USA, 1971–2000 vs. 2001–2050	2.3–3.4 (× 1.4)	Climate change	This study
Fort McCoy, WI, USA, 1971–2000 vs. 2051–2069	2.1–5.2 (× 2.2)	Climate change	This study
Vector-borne diseases
Dengue in Columbia	0.88–3.87 (× 4.4)	Human and mosquito density	Padmanabha et al. 2012
Dengue in Brazil	1.5–2.75 (× 1.8)	With and without adult mosquito control	Pinho et al. 2010
Dengue in Brazil	1.6–22.9 (× 14.3)	City and year	Degallier et al. 2009
Chikungunya in Italy	1.8–6.0 (× 3.3)	Local variations in mosquito abundance	Poletti et al. 2011
Leishmaniasis (*Leishmania infantum*) in dogs	5.9–11 (× 1.9)	Countries	Dye et al. 1992; Quinnell et al. 1997
Bluetongue virus	1.8–11 (× 6.1)	Geographic regions of the Netherlands	Santman-Berends et al. 2013
African horse sickness in zebra	10–23 (× 2.3)	Virus strain	Lord et al. 1997
Endoparasites
Nematodes of sheep	6–16 (× 2.7)	Nematode species	Kao et al. 2000
Oncherciasis	5.3–7.7 (× 1.5)	Countries	Filipe et al. 2005

The projected increases in *R*_0_ are equivalent, for the most part, to ranges of values of *R*_0_ estimated for other globally important parasites and pathogens associated with variations in major determinants of their ecology and epidemiology such as geographic location, pathogen genotype, different epidemics, reservoir host or vector density, and control efforts (Table 1).

## Discussion

These findings suggest that increasing temperatures in northern North America that support an *R*_0_ for *I. scapularis* of > 1.5 have been coincident with, or in advance of, but not subsequent to, expanding numbers of locations where *I. scapularis* populations have become established. In Canada, where we have tracked the spread of *I. scapularis*, temperature has remained a statistically significant determinant of *I. scapularis* occurrence in field studies and analyses of surveillance data that accounted for alternative environmental determinants (e.g., host abundance, altitude, rainfall, habitat types, tick immigration rates) (Bouchard et al. 2013a, 2013b; Leighton et al. 2012; Ogden et al. 2008; 2010). These observations supported a key role for temperature in *I. scapularis* populations becoming established at the northern edge of the tick’s range. Also, *I. scapularis* population expansion in Canada is occurring despite an overall deforestation (Natural Resources Canada 2013) rather than the reforestation thought to have driven the initial reemergence of Lyme disease in the United States (Barbour and Fish 1993). Forest fragmentation may enhance Lyme disease risk for a variety of reasons; however, *I. scapularis* ticks are invading Canada where forest fragmentation occurred over time scales long predating current *I. scapularis* invasion (Elliott 1998). Together, these findings suggest that even if recent warming in the region (5–10% increases in DD > 0°C; Figure 1) was not associated with global warming, a future warming climate will increase *R*_0_ of *I. scapularis* in northern North America. Increases in *R*_0_ may drive increased Lyme disease risk where it is already endemic (within limits determined by density-dependent regulation of the tick) and drive range expansion into more northern regions where it is currently absent. Furthermore, they support the hypothesis that climate warming in northeastern North America may have co-driven the emergence of Lyme disease risk, alongside other hypothesized factors such as reforestation and burgeoning deer populations (Wood and Lafferty 2013), by facilitating the spread of *I. scapularis* from refuges. Expansion of *I. scapularis* in the northern United States has then provided the source of ticks to fuel its northward expansion into Canada.

The immediate importance of future increased *R*_0_ of *I. scapularis* in the northeast and upper Midwest of North America is that *a*) regions currently climatically unsuitable become suitable for *I. scapularis* establishment (i.e., *R*_0_ changes from < 1 to > 1; Figure 2); *b*) in regions currently suitable for *I. scapularis* (where *R*_0_ > 1), tick invasion speed will accelerate as the likelihood of stochastic tick population fade-out reduces (May et al. 2001), and tick-borne pathogen invasion speed increases due to increasing tick abundance (Ogden et al. 2007); and *c*) risk from *I. scapularis*–transmitted pathogens may increase where the tick and pathogen are already established due to increased tick abundance up to a point at which this is limited by density-dependent regulation (Ogden et al. 2007). To date, *I. scapularis* invasion in the northern United States and Canada has been followed by invasion of the agent of Lyme disease, *B. burgdorferi* sensu stricto (Hamer et al. 2010; Ogden et al. 2013), hence we assume northward *I. scapularis* range expansion is synonymous with expansion in Lyme disease risk.

The magnitude of projected increases in *R*_0_ of *I. scapularis* in the present study is of importance for the ecology and epidemiology of vector-borne diseases in general. This was illustrated by *R*_0_ ranges estimated for other globally important parasites and pathogens associated with variations in known key determinants of their ecology and epidemiology (Table 1). These ranges were of a similar magnitude to projected increases in *R*_0_ of *I. scapularis* with climate change. Tick species are likely to respond to the central tendency of increasing temperatures due to *a*) the long periods of inter-stadial development that take place in the surface layers of the soil where fluctuating air temperature are buffered, *b*) latency in responses of development rates to temperature changes minimizing effects of very short-term temperature fluctuations (Ogden et al. 2004), *c*) their ability to return to soil-level refugia during extremes of heat, cold, drought, or rainfall while host seeking, *d*) their associations with woodland habitats within which a microclimate is buffered from extremes of temperature occurring in treeless areas (Lindsay et al. 1999b; Morecroft et al. 1998), and *e*) ticks have no nonparasitic immature feeding stages whose survival is susceptible to short-term changes in weather as do dipteran vectors such as mosquitoes. Therefore, the increases in *R*_0_ projected here represent a possible magnitude of increase in mean *R*_0_ values arthropod vectors may experience with climate change. Around this mean, dipteran population *R*_0_ may fluctuate seasonally and annually over a much wider range due to the rapid effects of rainfall and temperature on reproduction and mortality rates. Abundance and geographic distributions of many mosquito-borne diseases are currently driven primarily by control efforts that superimpose on any climate effects. Large increases in vector *R*_0_ may, however, render current vector and vector-borne disease control methods ineffective as vector multiplication outstrips control efforts (Massad, 2008; Reithinger et al. 2003; Smith et al. 2007).

*R*_0_ increases associated with climate change may be limited in some circumstances. Host population densities and habitat do not seem to be currently limiting on *I. scapularis* range expansion, but they may be in the future. Mosquitoes and other dipteran vectors can be dispersed by wind (Service 1997), but ticks need hosts for their dispersal to effect range expansion. *I. scapularis* are dispersed over long distances by migratory birds, but ticks that are not carried by migratory animals would be expected to have less capacity to invade climatically suitable environments. Ticks of public health importance such as *I. ricinus* and *I. scapularis* are mostly host and woodland habitat generalists, which facilitates range changes, whereas the more highly specialized the niche of a species, the less likely it will be to be dispersed and/or capable of becoming established in new locations (Morin and Chuine 2006).

## Conclusions

We estimated the degree to which projected climate change may impact the ecology of arthropod vectors and, by inference, vector-borne diseases. The emergence of Lyme disease in North America may itself have been partly driven by recent climate change. Confidence in our projections is increased by observed changes in temperature and estimated *R*_0_ for the vector associated with the actual emergence of the vector and the vector-borne diseases it transmits. Our findings suggest that effort should be refocused on assessing the health risks due to vector-borne disease, particularly vector-borne zoonoses, associated with our changing climate.

## Supplemental Material

(1.1 MB) PDF
