# Supplementary material for: Estimated Effects of Projected Climate Change on the Basic Reproductive Number of the Lyme Disease Vector Ixodes scapularis
Source: Environ Health Perspect. 2014 Mar 14;122(6):631–8. doi: 10.1289/ehp.1307799 (PMC4050516; doi:10.1289/ehp.1307799)
Supplement: (1.1 MB) PDF [file ehp.1307799.s001.pdf]

Environmental Health Perspectives doi:10.1289/ehp.1307799

Supplemental Material

Estimated Effects of Projected Climate Change on the Basic Reproductive Number of the Lyme Disease Vector *Ixodes scapularis*

Nicholas H. Ogden, Milka Radojević, Xiaotian Wu, Venkata R. Duvvuri, Patrick A. Leighton, and Jianhong Wu

Table of Contents **Page**

Variation in temperature and R_0 amongst Canadian sites **2**

Table S1. Basic characteristics of sites used in this study **4**

Figure S1. The locations of sites used in the study **5**

Figure S2. Model-estimated R_0 and DD > 0°C using observed daily temperature **5**

Figure S3. Climate classification tree for the sites **6**

Figure S4. Daily normal of ANUSPLIN daily maximum temperatures for each cluster **6**

Model sensitivity analysis **7**

Table S2. The parameters used in sensitivity analysis **9**

Fig S5. Local sensitivity analysis **10**

Fig S6. Results of global sensitivity analysis **11**

Validation of climate model output **11**

Table S3. Basic information about RCM simulations **13**

Figure S7. Mean DD > 0°C for each cluster using observed and model temperature **14**

Figure S8. Mean R_0 for each cluster using observed and climate model temperature **14**

Mapping R_0 **15**

Figure S9. Plots of model-estimated R_0 values plotted against DD > 0°C **16**

References **17**

Variation in temperature and R_0 amongst Canadian sites

Sites at which we estimated R_0 were selected in previous studies (Ogden et al. 2005; Wu et al. 2013) (Table S1) and form rough south-north transects in eastern Canada (Figure S1). They aimed to capture the range of climatic zones in this region (Natural Resources Canada 2013a), where *I. scapularis* is currently expanding its range, which is due to complex orography, water proximity and forest structure (Natural Resources Canada 2013b) that influence local temperature regimes. We re-investigated the degree of climate variability amongst these sites in order to come to a compromise between inter-site variability and the need for some simplification for data presentation. In earlier studies (Ogden et al. 2005) the best index of the suitability of seasonally-variable temperature, at a particular location, for *I. scapularis* survival was annual cumulative degree-days above 0°C (DD > 0°C) because temperature-dependent intersstadial tick development (a key variable through which temperature affects R_0) does not occur below 0°C (Ogden et al. 2004). We grouped the sites into five clusters on the basis of geographic proximity and climatological similarity. Both DD > 0°C from observed data and R_0 estimated ANUSPLIN data from 1971 to 2010 at the sites in each cluster are shown in Figure S2. The climatological similarity of sites within the clusters was supported by estimation of the Euclidian Distance of DD > 0°C calculated from ANUSPLIN data from 1971 to 2010 as a measure of similarity and Ward's criteria (Derry 2008; Munoz-Diaz and Rodrigo 2004; Romesburg 1990) for linking grid points with the highest similarity (Figure S3). The clusters were: 1) Southern Ontario - the north shore of Lake Erie, 2) Huron Ontario – Ontario adjacent to the south-east extent of Lake Huron, 3) Upper Southern Ontario – Ontario adjacent to the eastern extent of Lake Huron close to Georgian Bay, 4) South-Western Quebec, and 5) Boreal region - the most northern sites in both Ontario and Quebec. The significance of variations amongst clusters in the relationship between modelled R_0 and DD > 0°C was tested in a linear regression model (STATA SE11.0: Statacorp, College Station, Tx). R_0 estimated by the tick model was the outcome while DD > 0°C and clusters (as a categorical variable) were explanatory variables. Adjusted for DD > 0°C (coefficient = 2.1E-03, SE = 4.5E-05, P < 0.001), R_0 varied

significantly amongst clusters ($F = 163.4$, $df = 4$, $P < 0.001$) suggesting that presentation of simulations from all clusters separately would be more rigorous than combining all data. Differences amongst sites and clusters in the relationship between R_0 and DD > 0°C can be explained by differences in seasonal patterns of daily temperatures: R_0 is slightly higher in South-Western Quebec than Huron Ontario even though DD > 0°C values are similar because, despite longer winters (when temperatures are below 0°C), mid-summer temperatures in South-Western Quebec are higher resulting in faster tick development (Figure S4).

Table S1. Basic characteristics of sites used in this study.

Site number	Site	Location	Mean DD>0°C 1971-2000	Cluster name
**Ontario**				
1	Point Pelee	41°57'N 82°31'W	3791	S. Ontario
2	Chatham WCPC	42°23'N 82°12'W	3911	S. Ontario
3	New Glasgow	42°31'N 81°38'W	3536	S. Ontario
4	Port Stanley	42°40'N 81°13'W	3315	S. Ontario
5	Courtright	42°45'N 82°27'W	3734	S. Ontario
6	Delhi CDA	42°52'N 80°33'W	3441	S. Ontario
7	London Airport	43°02'N 81°09'W	3355	Huron Ontario
8	Exeter	43°21'N 81°29'W	3336	Huron Ontario
9	Blyth	43°43'N 81°23'W	3221	Huron Ontario
10	Hanover	44°07'N 81°00'W	3100	Upper S. Ontario
11	Wiarton Airport	44°45'N 81°06'W	2959	Upper S. Ontario
12	South Baymouth	45°35'N 82°01'W	2733	Upper S. Ontario
13	Timmins Airport	48°34'N 81°23'W	2351	Boreal region
14	Cochrane	49°04'N 81°02'W	2256	Boreal region
15	Kapuskasing CDA	49°24'N 82°26'W	2317	Boreal region
16	Smoky Falls	50°04'N 82°10'W	2283	Boreal region
**Quebec**				
17	Hemmingford	45°04'N 73°43'W	3076	S.W. Quebec
18	St Anicet	45°08'N 74°21'W	3167	S.W. Quebec
19	Iberville	45°20'N 73°15'W	3131	S.W. Quebec
20	Montreal McGill	45°30'N 73°35'W	3409	S.W. Quebec
21	Ste Thérèse Ouest	45°39'N 73°53'W	3000	S.W. Quebec
22	St Janvier	45°44'N 73°53'W	2860	S.W. Quebec
23	Fleury	45°48'N 73°00'W	2969	S.W. Quebec
24	Sorel	46°02'N 73°07'W	3095	S.W. Quebec
25	St Côme	46°17'N 73°45'W	2417	S.W. Quebec
26	St Zenon	46°37'N 73°52'W	2236	Boreal region
27	St Michel des Saints	46°41'N 73°55'W	2392	Boreal region
28	Grande Anse	47°06'N 72°56'W	2575	Boreal region
29	La Dore	48°46'N 72°43'W	2264	Boreal region
30	Chapais 2	49°47'N 74°51'W	2001	Boreal region

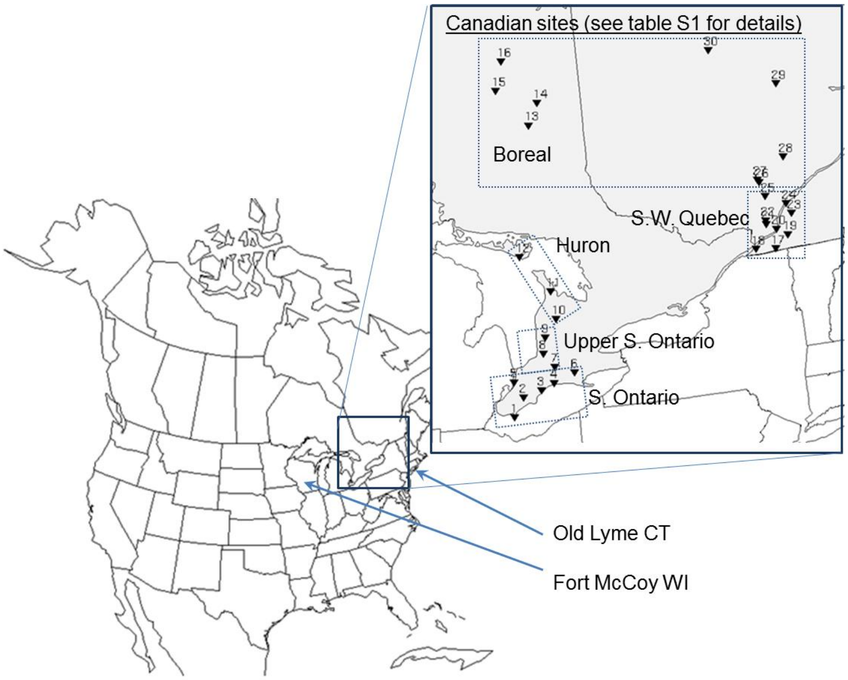

Figure S1. The locations of sites used in the study, including the locations of clusters of sites in Canada.

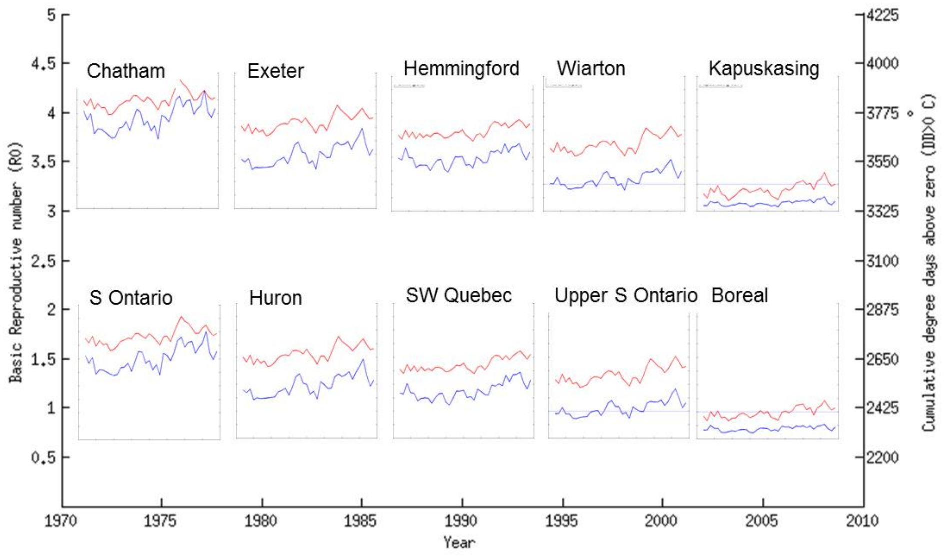

Figure S2. Model-estimated R_0 (left-hand Y-axis and blue lines) and DD > 0°C (right-hand Y-axis and red lines) for example sites from each cluster (upper panel), and the mean values for each cluster (lower panel), using observed daily temperature for 1971 to 2010. The axes surrounding the graphs are applicable to each graph in each panel.

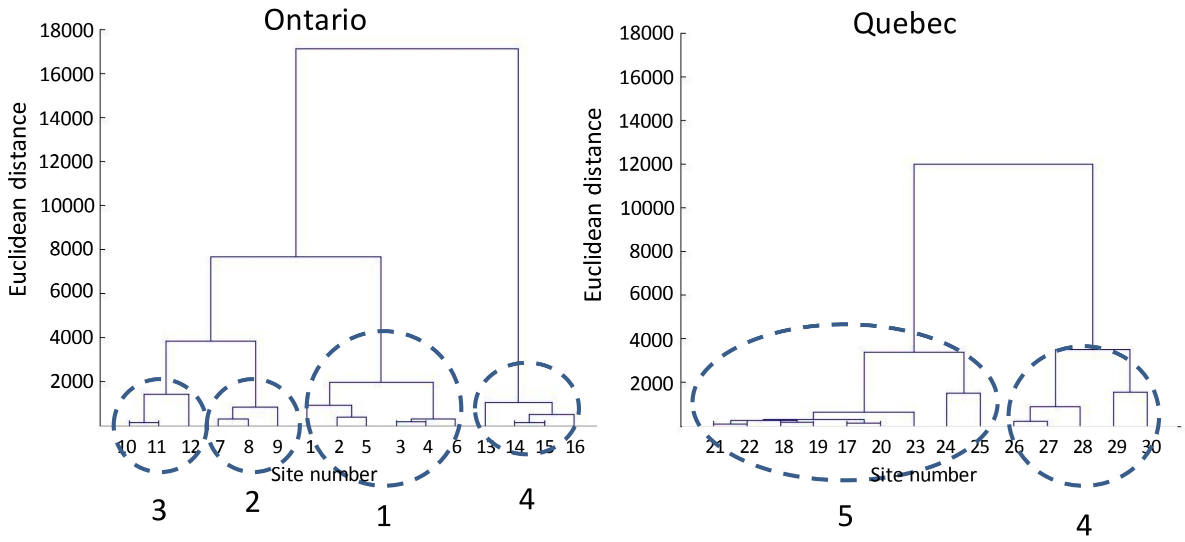

Figure S3. Climate classification tree for the sites based on 1971 to 2010 annual DD > 0°C, derived from ANUSPLIN temperature data. The X-axis shows station index (Table S1), while the numbered circles refer to the 'clusters' of sites: 1. Southern Ontario; 2. Huron; 3 Upper S. Ontario; 4. S.W. Quebec; and 5. Boreal region. The Y-axis is the Euclidian distance as a measure of climate similarity.

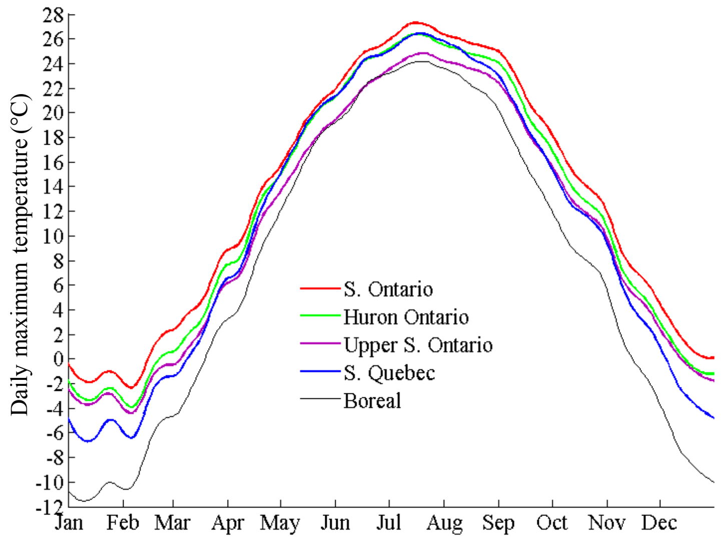

Figure S4. Daily normal of ANUSPLIN daily maximum temperatures for each cluster for the period 1971 to 2010.

Model sensitivity analysis

The *I. scapularis* model is sensitive to a greater or lesser degree to changes in model parameter values, particularly changes in temperature (which is explicitly modelled here), but also host abundance and off-host tick mortality (Wu et al. 2013). Host abundance (deer and mice in this model) will vary from location to location on both local and national scales depending on the specific suitability of habitat in each location for those species. Off-host tick mortality varies with the capacity of the environment of the surface layers of the soil, which provide the environment for ticks during the long period of development from one instar to the next, to protect the ticks from degrees of temperature, desiccation or flooding that result in greater mortality (Ogden et al. 2006). This results in many environments being unsuitable for tick survival, and partly explains the association between *I. scapularis* and woodland habitats. Woodland habitats do vary in their capacity to provide refugia for developing ticks, which is reflected in different mortality rates of ticks undergoing intersstadial development (Ogden et al. 2006). To assess the sensitivity of our projected changes in R_0 to all model variables we performed local and global sensitivity analyses. For these analyses, monthly temperature data for 1971-2070 obtained from the regional climate model CRCM 4.2.3 were used to determine the temperature-dependent tick intersstadial development rates and tick activity rates (Wu et al. 2013). All model variables were incorporated in the sensitivity analyses (Table S2). All baseline values were those in (Wu et al. 2013), and all simulations were performed in Matlab R2010a (MathWorks, Natick, MS, USA).

Local sensitivity analysis

Local sensitivity analysis was used to quantify the impact of variation of individual parameters on estimated R_0 (Benton and Grant 1999). Local sensitivity analyses were performed for data from one site of each of the five Canadian clusters: Point Pelee, London, Wiarton, Hemmingford and Timmins. All parameters were varied one at a time by 5% (above and below baseline values, with all other parameter values set at baseline values) and 3-year mean R_0 values for each site for each year from 1972 to 2069

were obtained. A comparative index S^i was used to assess the sensitivity of the change in R_0, from one time slice to another, to a 5% change in the ith parameter:

$$S^i=(r^i_2-r^i_1)-(r^i_{20}-r^i_{10}),$$

where r^i_2 is the average value for R_0 for the later time period, and r^i_1 is the average value for R_0 for the earlier time period each of the periods 1972-2000, 2001-2050 and 2051-2069, when the ith parameter under investigation increases or decreases by 5%. The average R_0 for the same time periods using baseline values for all model parameters are r^i_{20} for the later time period and r^i_{10} for the earlier time period. This analysis indicated that the degree of change in R_0 from one time period to the next was most sensitive to development rates and mortality rates of eggs and larvae (Fig S5), although the effects of changing these parameter values by 5% had relatively modest effects on increases in R_0 from one time period to another (changes of 0.15 or less) (Fig S5). Simultaneously changing mortality rates of all off-host tick stages by 5% (as would be expected to occur in different woodland types: Ogden et al. 2006) had a larger effect on increases in R_0 (changes of 0.28 or less) from one time period to another (Fig S5).

Global sensitivity analysis

We used Latin Hypercube sampling (LHS) to estimate the possible range of R_0 caused by simultaneous variation of multiple parameters. LHS is an efficient and widely used statistical sampling method of uncertainty analysis that treats each parameter as a random variable within a pre-determined range (Matser et al. 2009; Marino et al. 2008). In this global sensitivity analysis, we considered only one site (Point Pelee) due to the high computer capacity required. The pre-determined range of values of parameters was between 20% above and below baseline values (Matser et al. 2009). After creating 1000*18 parameter space using Latin Hypercube sampling with uniform distribution (using the LHS package), we estimated 1000 values of R_0 at Point Pelee for each year from 1971-2070 by running 1000 simulations. The 95% confidence intervals and minimum and maximum values for R_0 obtained in the LHS are shown in Fig S8. This analysis showed that the 95% confidence intervals are close to the

baseline values and that increases in values of R_0 estimated by the model with projected increasing climate are obtained with a wide range of parameter values.

Table S2. The parameters used in sensitivity analysis, and the ranges of parameter values used in global sensitivity analysis.

**Parameters under investigation**	**Range of parameters for Latin Hypercube Sampling (LHS)**
Number of rodents	U(200*0.8, 200*1.2)
Number of deer	U(20*0.8, 20*1.2)
Density-dependent effect on fecundity	U(0.0104*0.8, 0.0104*1.2)
Duration of hardening of larvae	U(1/21*0.8, 1/21*1.2)
Development rate of fed larvae	U(1/3*0.8, 1/3*1.2)
Development rate of fed nymphs	U(1/5*0.8, 1/5*1.2)
Development rate of fed adult females	U(1/10*0.8, 1/10*1.2)
Eggs mortality	U(0.002*0.8, 0.002*1.2)
Hardening larvae mortality	U(0.006*0.8, 0.006*1.2)
Questing larvae mortality	U(0.006*0.8, 0.006*1.2)
Density-dependent mortality of feeding larvae	U(0.3909*0.8, 0.3909*1.2)
Engorged larvae mortality	U(0.003*0.8, 0.003*1.2)
Questing nymphs mortality	U(0.006*0.8, 0.006*1.2)
Density-dependent mortality of feeding nymphs	U(0.2909*0.8, 0.2909*1.2)
Engorged nymphs mortality	U(0.002*0.8, 0.002*1.2)
Questing adults mortality	U(0.006*0.8, 0.006*1.2)
Density-dependent mortality of feeding adult females	U(0.3537*0.8, 0.3537*1.2)
Engorged adult females mortality	U(0.001*0.8, 0.001*1.2)

Note that U(--,--) indicates the uniform distribution in the process of allocation using LHS.

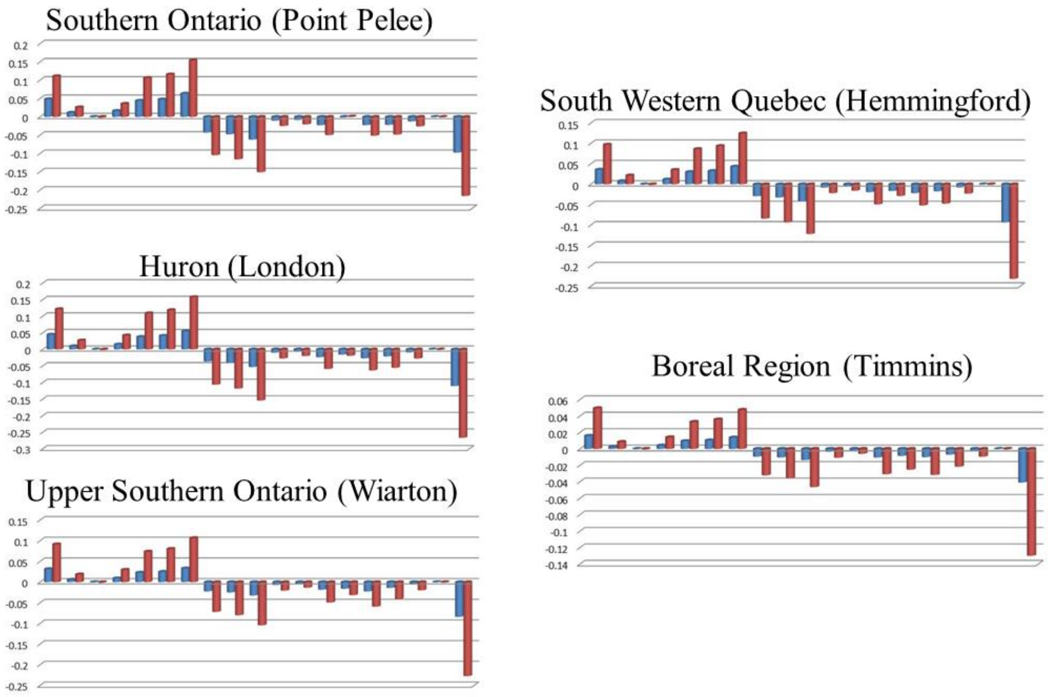

Figure S5. Local sensitivity analysis indicating the relative sensitivity (S^i on the Y-axis) of increases in R_0 from the periods 1972-2000 to 2001-2050 (blue bars) and from 1972-2000 to 2051-2069 (red bars), to 5% increases in individual parameter values. The parameters were (from left to right in each graph and in the same order as in Table S2) numbers of deer and rodents, density-dependent effects on fecundity, duration of larval hardening, development rates of larvae, nymphs and adults, mortality rates of eggs, and hardening and questing larvae, density dependent mortality of feeding larvae, engorged larva and questing nymph mortality, density-dependent mortality of feeding nymphs, mortality of engorged nymphs and questing adults, density-dependent mortality of feeding adults and mortality rates of engorged adults. The final bars show the combined effects of changes in mortality rates of all off-host ticks simultaneously.

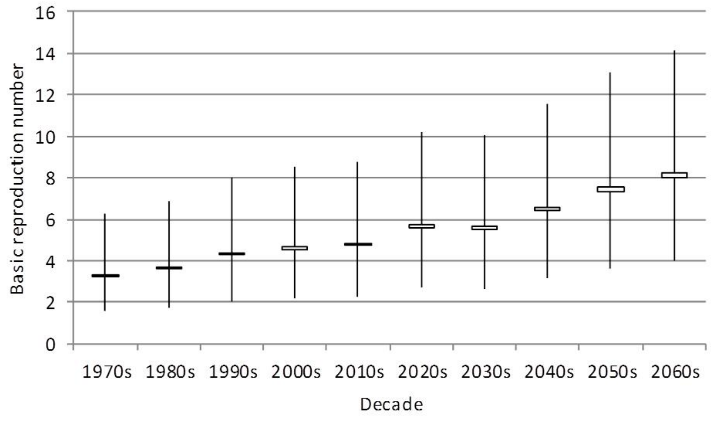

Figure S6. Results of global sensitivity analysis by Latin Hypercube sampling. The 95% confidence intervals for values for R_0 are shown by the boxes while the whiskers indicate the maximum and minimum values obtained.

Validation of climate model output

To validate CRCM4.2.3 temperature output used in modelling R_0, and to compare with an ensemble of climate models (Table S3), we compared DD > 0°C output from the models with observed (ANUSPLIN) data. In addition to CRCM4.2.3 (Laprise et al. 1997; Music et al. 2007), two RCM runs were available from the North American Regional Climate Change Assessment Program (NARCCAP; Mearns et al. 2012): Weather Research Forecast (WRF: Skamarock et al. 2005) and Mesoscale Model (MM5I: Grell et al. 1993). All RCMs were driven by initial and boundary conditions supplied by GCM output as used in the Intergovernmental Panel on Climate Change (IPCC) Fourth Assessment Report (IPCC 2007). Initial and boundary conditions of CGCM3.1 T47 (McFarlane et al. 2005; Scinocca et al. 2008) drove CRCM4.2.3 and WRF runs, and those of CCSM3 (Collins et al. 2006) were drivers for MM5I but also for WRF. In both CGCM3.1 T47 and CCSM3, until year 2000, green-house gas emissions were as in the CMIP 20th century experiment (Meehl et al. 2000). Future projections, starting in 2001, follow the IPCC Special Report on Emission Scenarios (SRES: http://www.ipcc.ch/pdf/special-reports/spm/sres-en.pdf) A2 emission scenario (Nakicenovic and Swart 2000). The A2 scenario, which is described as mid-high

Green-House-Gas (GHG) emission scenario with continuously increasing global population and regionally oriented economic growth, was chosen because of the availability of RCM model output using this emissions scenario. Daily temperatures from output of each RCM and GCM were aggregated onto common grid projection with ANUSPLIN at approximately 45 km horizontal grid resolution, true at 60°N (Heum et al. 2012). DD > 0°C was computed from each model output over a common time window for each site for periods of past climate from 1971 to 1999 and future climate from 2041 to 2069. This analysis showed that CGCM3.1 and those RCMs driven by CGCM3.1 under-estimate temperature, while MM5I driven by CCSM3 significantly over-estimates temperature in warmer regions of such as southern Ontario . On the other hand, WRF driven by CCSM3 shows reasonable agreement with ANUSPLIN. As a consequence of these observations, the mean bias correction method (Watanabe et al. 2012) relative to monthly temperature from ANUSPLIN was applied to CRCM4.2.3 to improve the accuracy of temperature prediction (Fig S7) and bias-corrected temperature data were used in estimations of R_0 by the *I. scapularis* model. R_0 estimates for the Canadian sites using observed temperature data and climate models output are presented in Fig S8.

Table S3. Basic information about RCM simulations and their corresponding GCM used to drive atmospheric initial and boundary conditions. The temperature dataset obtained from WRF and MM5I were sourced through the North American Regional Climate Change Assessment Program NARCCAP project (NARCCAP 2012)

Acronym	Name	Institution	Reference	Driving conditions	Horizontal resolution
CRCM4.2.3	Fourth version of Canadian RCM	Canadian Regional Climate Modelling and Diagnostic (CRCMD) Network, Canada	Laprise et al. 1997; Music and Caya. 2007	CGCM3.1 T47	45 km true at 60°N
WRF	Weather Research Forecast	A collaborative partnership* USA (WRF 2013),	Skamarock et al. 2005	CGCM3.1 T47, CCSM3	0.5° latitude-longitude
MM5I	Mesoscale Model	Iowa State University/NCAR, USA (NCAR 2013a)	Grell et al. 1993	CCSM3	
CGCM3.1	Third version of Canadian GCM	Canadian Centre for Climate Modelling and Analysis (CCCma), Environment Canada, Canada (Environment Canada 2013)	McFarlane et al. 2005; Scinocca et al. 2008	Not applicable	approximately 3.75° latitude-longitude
CCSM3	Third version of Community Climate System Model	National Center for Atmospheric Research, USA (NCAR 2013b),	Collins et al. 2006	Not applicable	approximately 1.4° latitude-longitude

*National Center for Atmospheric Research (NCAR), the National Oceanic and Atmospheric Administration (the National Centers for Environmental Prediction (NCEP) and the Forecast Systems Laboratory (FSL), the Air Force Weather Agency (AFWA), the Naval Research Laboratory, the University of Oklahoma, and the Federal Aviation Administration (FAA).

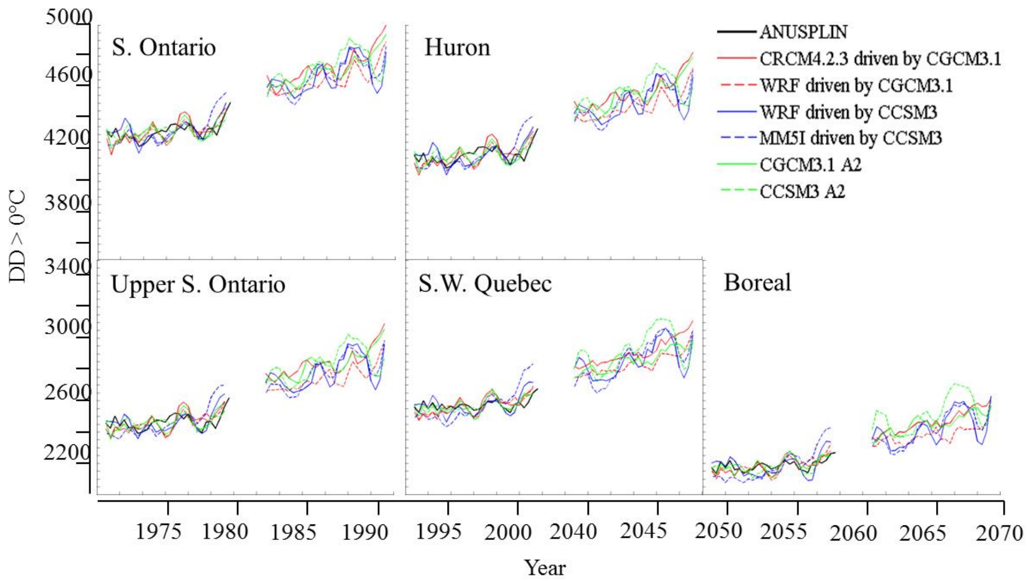

Figure S7. Mean DD > 0°C for each cluster of sites, calculated from observed ANUSPLIN temperature data (black line), and bias-corrected output from three RCM and two GCM simulations (coloured lines) for the time slices 1971 to 1999 and 2041 to 2069. The axes surrounding the graphs apply to each individual graph.

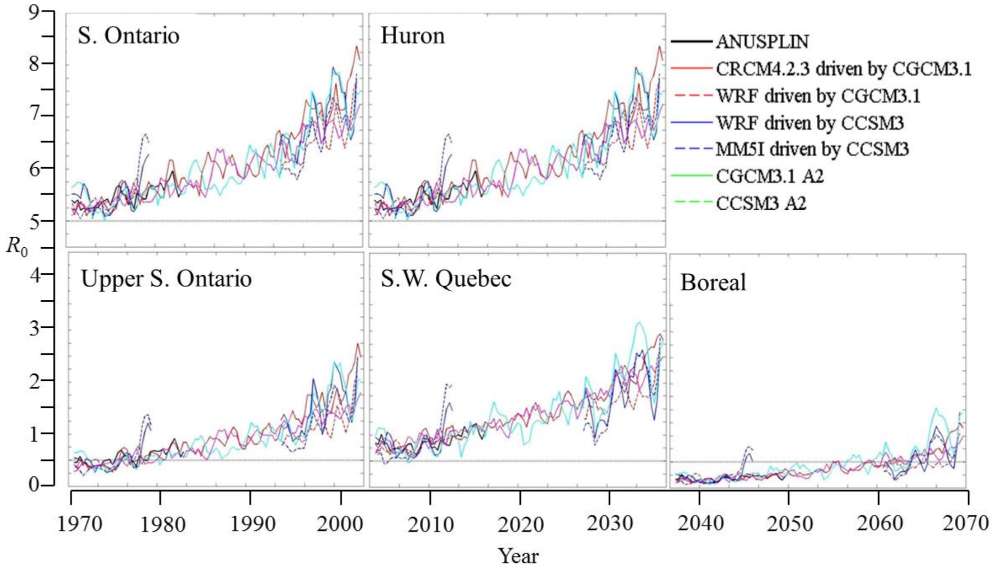

Figure S8. Annual estimates of mean R_0 for each cluster obtained using observed ANUSPLIN temperature data (black line) for 1972 to 2009, and bias-corrected temperature output from three RCM and two GCM simulations (colored lines) for 1972 to 2069. The axes surrounding the graphs apply to each graph.

Mapping R_0

In order to map R_0 under current and projected future climate we used our model-estimated values for R_0 under recent climate for the 30 Canadian sites over 40 years of observed temperature data (1971-2010) to obtain a relationship between R_0 and DD > 0°C. We only have R_0 values for the study sites but we have current and future projected DD > 0°C data for most of North America. Using the 'Basic fitting' tool of Matlab® R2010a (MathWorks, Natick, MS, USA), we obtained two regression functions that described the relationship between R_0 and DD > 0°C for each year from 1971 to 2010 (Fig S9, top panel): i) a linear function $R_{0L} = a \cdot DD > 0 + c$ where $a = 0.002367$ and $c = -7.1206$; or ii) a quadratic function $R_{0Q} = a \cdot DD > 0 + b \cdot\ DD > 0^2 + c$ where $a = 1.0717E - 06$, $b = -0.0046577$ and $c = 5.5555$. We selected the best fit relationship based on the coefficient of the determination (R^2) and the Root Mean Square Difference (RMSD). For the linear relationship, $R^2 = 0.9053$ and RMSD = 0.2305 while for the quadratic relationship $R^2 = 0.9184$ and RMSD = 0.2135. The linear relationship under-estimates R_0 for the cold and warm tails of annual temperature regimes, while the quadratic function shows the opposite behavior (Fig S9, bottom panel). However due to the better predictive power of the quadratic function, this was chosen for mapping purposes.

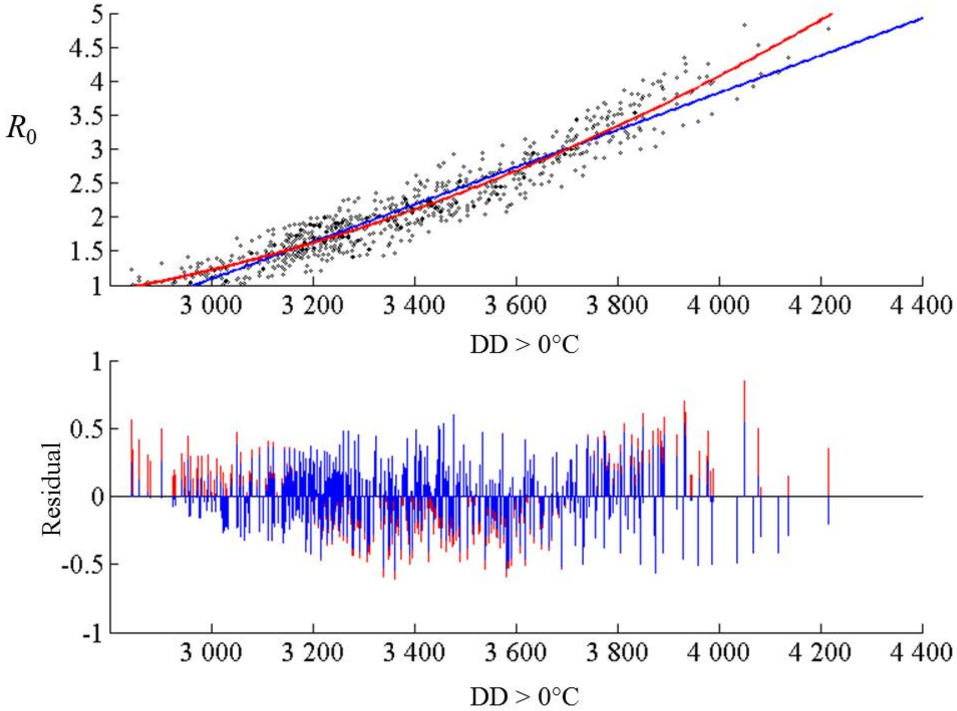

Figure S9. A scatter-plot (top panel) and plot of residuals (bottom panel) of model-estimated R_0 values plotted against DD > 0°C for each of the 30 sites for each year from 1971 to 2010. Temperature data used in the model were observed ANUSPLIN temperature data. The best fit relationships (top panel) and residuals (bottom panel) are shown in blue for the linear relationship and red for the quadratic relationship.

References

Benton TG, Grant A. 1999. Elasticity analysis as an important tool in evolutionary and population ecology. Trends Ecol Evol 14(12):467-471.

Collins WD, Bitz CM, Blackmon ML, Bonan GB, Bretherton CS, Carton JA et al. 2006. The Community Climate System Model version 3 (CCSM3). J Climate 19(11):2122–2143. doi: http://dx.doi.org/10.1175/JCLI3761.1

Derry JE. 2008. Regional patterns of snow cover water equivalent in the Colorado river basin using snowpack telemetry (SNOTEL) data. Master of Science Thesis (Colorado State University, Colorado), 92pp.

Environment Canada 2013. The Third Generation Atmospheric General Circulation Model. Available: http://www.ec.gc.ca/ccmac-cccma/default.asp?lang=En [accessed 6 December 2013].

Grell G, Dudhia J, Stauffer DR. 1993. A description of the fifth generation Penn State/NCAR Mesoscale Model (MM5), Ncar Tech Note NCAR/TN-398 (NCAR, Boulder), 107pp.

Heum H-I, Gachon P, Laprise R, Ouarda T. 2012. Evaluation of regional climate model simulations versus gridded observed and regional reanalysis products using a combined weighting scheme. Clim Dyn 31(7-8):1422-1457. doi : 10.1007/s00382-011-1149-3

IPCC. 2007. Climate Change 2007: The Physical Science Basis. Contribution of Working Group I to the Fourth Assessment Report of the IPCC, eds Solomon S et al., (Cambridge University Press, Cambridge), 996 pp.

Laprise R, Caya D, Bergeron G, Giguère M. 1997. The formulation of André Robert MC2 (Mesoscale Compressible Community) model. Atmos-Ocean 35(Suppl 1):195-220.

Marino S., Hogue IB, Ray CJ, Kirschner DE. 2008. A methodology for performing global uncertainty and sensitivity analysis in systems biology. J Theor Biol 254(1):178–196. doi: 10.1016/j.jtbi.2008.04.011.

Matser A, Hartemink N, Heesterbeek H, Galvani A, Davis S. 2009. Elasticity analysis in epidemiology: an application to tick-borne infections. Ecol Lett 12(12):1298–1305. doi: 10.1111/j.1461-0248.2009.01378.x.

McFarlane NA Scinocca JF, Lazare M, Harvey R, Verseghy D, Li J. 2005. The CCCma third generation atmospheric general circulation model. CCCma, Internal Report, 25 pp.

Mearns LO, Arritt R, Biner S, Bukovsky MS, McGinnis S, Sain S et al. 2012. North American climate change assessment program: Overview of phase I results. Bull Amer Meteor Soc 93(9):1337-1362.

Meehl GA, Boer GJ, Covey C, Latif M, Stouffer RJ. 2000. The coupled model intercomparison project (CMIP). Bull Amer Meteor Soc 81(2):313–318.

Munoz-Diaz D, Rodrigo FS. 2004. Impacts of the North Atlantic Oscillation on the probability of dry and wet winters in Spain. Clim Res 27(1):33-43.

Music B, Caya D. 2007. Evaluation of the hydrological cycle over the Mississippi river basin as simulated by the Canadian Regional Climate Model (CRCM). J Hydrometeorol 8(5):969-988.

Nakicenovic N and Swart R. 2000. Special Report on Emissions Scenarios: A Special Report of Working Group III of the Intergovernmental Panel on Climate Change. (Cambridge University Press, Cambridge), 599 pp.

NARCCAP 2012. North American Regional Climate Change Assessment Program project. Available: http://www.narccap.ucar.edu/ [accessed 6 December 2013].

Natural Resources Canada 2013a. Regional, national and international climate modeling. Available: http://cfs.nrcan.gc.ca/projects/3/3 [accessed 6 December 2013].

Natural Resources Canada 2013b. Forests in Canada: classification. Available: http://cfs.nrcan.gc.ca/pages/125 [accessed 6 December 2013].

NCAR 2013a. National Centre for Atmospheric Research. MMM: Mesoscale & Microscale Meteorology Division. Available: http://www.mmm.ucar.edu/ [accessed 6 December 2013].NCAR 2013b. National Centre for Atmospheric Research. Available: http://ncar.ucar.edu/ [accessed 6 December 2013].

Ogden NH, Lindsay LR, Beauchamp G, Charron D, Maarouf A, O'Callaghan CJ et al. 2004. Investigation of relationships between temperature and developmental rates of tick *Ixodes scapularis* (Acari: Ixodidae) in the laboratory and field. J Med Entomol 41(4):622-633.

Ogden NH, Bigras-Poulin M, O'Callaghan CJ, Barker IK, Lindsay LR, Maarouf A et al. 2005. A dynamic population model to investigate effects of climate on geographic range and seasonality of the tick *Ixodes scapularis*. Int J Parasitol 35(4):375-389.

Ogden NH, Barker IK, Beauchamp G, Brazeau S, Charron DF, Maarouf A et al. 2006. Investigation of ground level and remote-sensed data for habitat classification and prediction of survival of *Ixodes scapularis* in habitats of southeastern Canada. J Med Entomol 43(2):403-414.

Romesburg HC. 1990. Cluster Analysis for Researchers, (Robert E. Krieger Publishing, Malabar), 330pp.

Scinocca JF, McFarlane NA, Lazare M, Li J, Plummer D. 2008. The CCCma third generation AGCM and its extension into the middle atmosphere. Atmos Chem Phys Discuss 8(2):7883-7930.

Skamarock WC, Klemp JB, Dudhia J, Gill DO, Barker DM, Wang W, Powers J. 2005. A description of the advanced Research WRF version 2. NCAR Tech. Note NCAR/TN-468+STR, (NCAR, Boulder), 88pp.

Watanabe S, Kanae S, Seto S, Yeh, PJ-F, Hirabayashi Y, Oki T. 2012. Intercomparison of bias-correction methods for monthly temperature and precipitations as simulated by multiple climate models. J Geophys Res-Atmos 117:D23114. doi: 10.1029/2012JD018192.

WRF 2013. The Weather Research & Forecasting Model. Available: http://www.wrf-model.org/index.php [accessed 6 December 2013].

Wu X, Duvvuri VR, Lou Y, Ogden NH, Pelcat Y, Wu J. 2013. Developing a temperature-driven map of the basic reproductive number of the emerging tick vector of Lyme disease *Ixodes scapularis* in Canada. J Theor Biol 319:50-61. doi: 10.1016/j.jtbi.2012.11.014.
